# Rare diseases: a challenge for medicine and public health

**DOI:** 10.25646/11826

**Published:** 2023-12-13

**Authors:** Angela Fehr, Franziska Prütz

**Affiliations:** 1 Robert Koch Institute, Berlin, Centre for International Health Protection (ZIG); 2 Robert Koch Institute, Berlin, Department of Epidemiology and Health Monitoring

This issue of the Journal of Health Monitoring is dedicated to a public health topic for which population-wide health monitoring can hardly provide any data: rare diseases, their research and the care of those affected.

Rare diseases are also referred to as ‘orphan diseases’ and their medications as ‘orphan drugs’. These terms are used by those affected and researchers to describe the fact that they are orphans who have received little attention in research and healthcare. The prevalence of the individual, approximately 8,000 known rare diseases may be low, but the total population of those affected is not. In the European Union there are approximately 30 million people living with a rare disease; in Germany there are an estimated 4 million. Worldwide, it is estimated that 300 million people are affected [1].

Rare diseases are predominantly diseases of genetic origin with often severe, chronic courses. There are no diagnostic procedures or therapeutic guidelines for many rare diseases, and available treatments often only alleviate symptoms but cannot cure the disease. In a 2005 survey of 5,980 patients, the European Organisation for Rare Diseases (EURORDIS) found that 25 % had waited between 5 and 30 years for a correct diagnosis, 40 % of respondents had been misdiagnosed at the onset of the disease, which led to incorrect medication in 33 %, surgery due to misdiagnosis in 16 % and inappropriate psychological counselling in 10 % [2, 3]. As a result, there is a lack of comprehensive expertise and seamless patient pathways.

The development of drugs for rare diseases also entails particular challenges. The pharmaceutical industry expects high costs but low revenues due to small case numbers. The ‘orphaning’ of drug development for rare diseases can be traced back to regulatory progress: as a result of the thalidomide tragedy, legislative changes were passed in the USA in 1962, according to which controlled studies must be carried out to prove the safety and efficacy of all drugs. Drug safety increased, while costs rose at the same time. Research and development of drugs for a small number of patients was postponed in favour of drugs for common diseases [4]. In the 1970s, patients with rare diseases in the USA joined forces, demanded the right to equal medical care and treatment and called for political action. Their commitment led to the passing of the first legislation for drugs for rare diseases, the U.S. Orphan Drug Act of 1983, which primarily contained financial incentives for the pharmaceutical industry. For the first time in the USA, this law defined rare diseases by their frequency (maximum 7 in 10,000 people). Laws with similar incentives but slightly different prevalence rates followed (Japan 1997: 4/10,000; Australia 1998: 1.1/10,000; EU 2000: 5/10,000) [5]. Recent studies identified orphan drug regulations in 92 out of 200 countries or regions analysed [6].

The heterogeneity of rare diseases, which encompass all medical disciplines, has long pushed rarity into the background as a common denominator and as the cause of the particular infrastructural problems [7]. This affects both research and the challenges in healthcare provision. In their article, Schlangen and Heuing provide an overview of developments in the healthcare situation for rare diseases in Germany and describe goals, successes, and challenges. The National Action League for People with Rare Diseases (NAMSE), which was founded in 2010 on the initiative of the Federal Ministry of Health (BMG), the Federal Ministry of Education and Research (BMBF), the Alliance for Chronic Rare Diseases (ACHSE), a network of self-help organisations for people with rare diseases, and 25 other alliance partners, plays an important role in this. In 2013, NAMSE published a National Plan of Action for People with Rare Diseases. According to a report published in 2023 [8], establishing centres for rare diseases at three different levels (reference centres, centres of expertise and cooperating centres for a specific rare disease) as part of the action plan has already brought about significant improvements in healthcare.

A mapping of research into rare diseases from 2014 [9] shows that most of the projects were funded by the German Research Foundation (DFG) and the BMBF. Two thirds of the projects concerned disease-orientated basic research. In particular, rare oncological and haematological diseases were researched, another research area was rare genetic diseases, especially neurological diseases [9]. Since 2003, the BMBF has been funding networks that jointly research the causes and therapeutic approaches for rare diseases at various university locations. The patient registers often included in these networks provide the necessary data for clinical and healthcare research. Three research projects presented in this issue are examples of how complex the topic of rare diseases is and how important innovation and interdisciplinary and multinational cooperation are. Dutzmann et al. present the internationally networked cancer predisposition syndrome registry for the prevention and early detection of genetic alterations associated with an increased risk of cancer. Stapornwongkul et al. report on the GAIN registry (German genetic multi-organ Auto-Immunity Network), which records people with multi-organ autoimmune diseases in Germany, Italy and Portugal on the basis of a platform provided by the European Society for Immunodeficiencies (ESID). Endlich’s contribution describes a research project on a rare kidney disease (FSGS): this utilises a Nobel Prize-winning microscopy technique for the quantitative analysis of kidney tissue as well as high-throughput screening using zebrafish larvae for the rapid identification of potentially suitable drugs for treatment.

International research cooperation and sustainable funding are key to improving the diagnosis and treatment of rare diseases, especially in low- and middle-income countries. In 2019, Rare Disease International (RDI) signed a Memorandum of Understanding with the WHO and founded the Global Network for Rare Diseases (GNRD), based on the United Nations Declaration on Universal Health Coverage. The International Rare Diseases Research Consortium (IRDiRC) aims to ensure that all patients affected by a known rare disease receive the correct diagnosis within one year by 2027. European cooperation on rare diseases takes place, for example, in the European Joint Programme on Rare Diseases (EJP RD).

This issue of the Journal of Health Monitoring cannot cover the entire spectrum of topics relating to rare diseases, but aims to provide an insight into the many challenges and the special efforts being made to alleviate the suffering of many people with rare diseases.
